# A novel NEDD8-binding protein modulates NF-κB signaling pathway

**DOI:** 10.1186/ar3601

**Published:** 2012-02-09

**Authors:** Osamu Takashima, Fuminori Tsuruta, Manato Ebina, Yu Kigoshi, Shingo Nakamura, Tomoki Chiba

**Affiliations:** 1 Graduate School of Life and Environmental Sciences, University of Tsukuba, Tennodai, Tsukuba, Ibaraki 305-8577, Japan

## 

In canonical NF-κB signaling pathway, a ubiquitin ligase called SCF (Skp1, Cul1, F-box protein) complex is essential for I-κB degradation. The activity of the SCF complex is positively regulated by a post-translational modification of Cul1 subunit with a ubiquitin-like protein NEDD8. Like ubiquitin, NEDD8 possesses evolutionary conserved Lys residues on its surface, and forms poly-NEDD8 chain *in vivo *and *in vitro *[1,2]. Despite the importance of the NEDD8 modification in all eukaryotic cells, little is known about the function of poly-NEDD8 chain. To elucidate the function of the poly-NEDD8 chain *in vivo*, we screened poly-NEDD8 chain binding proteins (PNBPs) using a yeast two-hybrid system. Of the identified PNBPs, PNBP1 was identical to a gene present in non-HLA celiac disease and rheumatoid arthritis risk loci [3].

PNBP1 interacted with NEDD8, NEDD8-conjugating enzyme Ubc12 and Cul1. PNBP1 strongly associated with wild-type Cul1, but not its NEDDylation defective Cul1(K720R) mutant, suggesting that the interaction is mediated in part through NEDD8. Furthermore, PNBP1 promoted NEDDylation of Cul1 in an *in vitro *reconstitution assay. These activities were dependent on RING-finger domain of PNBP1. Finally, knockdown of PNBP1 led to reduction of the NF-κB activation, suggesting that PNBP1 is an important modulator of the NF-κB signaling pathway.

## References

[B1] Jones J Wu K Yang Y Guerrero C Nillegoda N Pan ZQ Huang L A targeted proteomic analysis of the ubiquitin-like modifier NEDDD8 and associated proteins J Proteome Res 2008 7 1274 1287 10.1021/pr700749v 18247557 PMC2676899

[B2] Ohki Y Funatsu N Konishi N Chiba T The mechanism of poly-NEDD8 chain formation *in vitro* Biochem Biophys Res Commun 2009 381 443 447 10.1016/j.bbrc.2009.02.090 19245792

[B3] Alexandra Zhernakova A Meta-analysis of genome-wide association studies in celiac disease and rheumatoid arthritis identifies fourteen non-HLA shared loci PloS Genet 2011 7 e10020004 10.1371/journal.pgen.1002004 PMC3044685 21383967

